# IMPACT: Integrated Model For Patient Acuity, Care, And Triage Efficiency In Emergency Department

**DOI:** 10.1007/s10916-026-02420-2

**Published:** 2026-09-23

**Authors:** Hamidreza Rasouli Panah, Abtin Ijadi Maghsoodi, Samaneh Madanian, Jian Yu

**Affiliations:** 1 Department of Computer and Information Sciences, Auckland University of Technology, Auckland, New Zealand; 2 Division of Health, Engineering, Computing and Science, University of Waikato, Hamilton, New Zealand

**Keywords:** Emergency department, Adverse outcome, Mortality, Length of stay, Waiting time, Health inequities

## Abstract

Adverse patient outcomes in Emergency Departments (ED) are shaped by a complex interplay of clinical, demographic, and operational factors, yet existing approaches often fail to account for these dimensions in an integrated and actionable manner. This study introduces the Integrated Model for Patient Acuity, Care, and Triage Efficiency (IMPACT), a comprehensive framework developed to identify key predictors of mortality and generate individual adverse outcome risk scores across multiple clinical timeframes using a retrospective analytical approach. A retrospective analysis was conducted using 593,673 de-identified ED presentations from a tertiary hospital in the Waikato region in New Zealand, between 2016 and 2024. The modelling strategy involved a two-step approach, starting with Generalised Linear Models (GLM) for inferential analysis, followed by multiple regularised logistic regressions to enhance predictive accuracy and mitigate multicollinearity. The results indicated that advanced age, greater clinical complexity, and longer ED stays were associated with increased adverse outcomes, while longer waiting times were inversely associated with adverse outcomes, a finding consistent with triage-induced prioritisation effects whereby the most acutely unwell patients are seen first and also face the highest mortality risk, an associational pattern rather than a causal relationship. Based on the final model, an adverse outcome scoring system was derived, translating patient-level data into individual probability scores. The IMPACT model demonstrated high discriminative performance, with AUROC values of 0.9532 for ED mortality, 0.9183 for 10-days mortality, and 0.8999 for 28-days mortality. By combining robust statistical inference with a clinically interpretable scoring system, IMPACT provides a scalable and practical tool for retrospective risk stratification, service evaluation, and equitable decision-making in emergency care settings.

## Introduction

Emergency Departments (EDs) are critical parts of healthcare systems, serving as the frontline for managing patients with diverse and urgent medical needs [1]. The fast-paced ED environment significantly influences clinical decision making and patient outcomes during their ED stay and in the subsequent patient’s journey [2]. This impact results from a complex interaction of demographic characteristics, clinical conditions, triage processes, and resource allocation efficiency [3–5]. Understanding how these factors interact to affect patient outcomes is crucial for improving care quality and optimising resource utilisation in emergency settings.

Among these factors, overcrowding and prolonged Length of Stay (LoS) in EDs are particularly pressing issues [6]. These phenomena frequently result in delayed medical care, compromised patient safety, and increased provider stress [7, 8], ultimately contributing to elevated morbidity and mortality rates [9, 10]. However, the challenge extends beyond these operational metrics. A broader array of determinants, including triage accuracy, patient condition complexity, demographic disparities, and continuity of care, collectively influence both short-term and long-term outcomes [11–14]. This complexity necessitates an integrated approach to understanding and addressing these interrelated factors, thereby improving overall patient outcomes.

To address the existing challenges, various approaches have been explored. A comprehensive review has analysed potential solutions and challenges across the health system, focusing on ED patient flow [1]. Another study investigated factors contributing to increased LoS, emphasising the importance of effective resource allocation and process optimisation [15]. Since 2009, the implementation of the “Shorter Stays in Emergency Departments” health target in New Zealand has demonstrated positive outcomes, including reduced adverse outcomes and enhanced ED efficiency [16]. However, these initiatives have not fully resolved the persistent challenges, particularly those related to demographic disparities and continuity of care. For example, it has been demonstrated that Māori experience higher mortality and re-presentation rates compared to non-Māori populations, highlighting persistent inequities in acute care settings that need targeted intervention [17]. This study further builds on such evidence by explicitly analysing outcome disparities among Māori and Pacifica populations within a large regional dataset. By quantifying inequities through a unified risk-scoring approach, the framework provides actionable insights for equitable service planning and culturally responsive interventions.

Despite the existing contributions, a significant gap remains in the literature concerning integrative analyses of the combined factors influencing patient outcomes. Current research often focuses on isolated factors, failing to capture their synergistic impact on short- and long-term outcomes. This limitation is particularly evident in studies analysing ED performance, where the complex interactions between operational metrics, clinical factors, and patient demographics remain poorly understood. The lack of comprehensive analysis delays the development of effective interventions aimed at improving care quality and reducing adverse events.

This study aims to evaluate the multifactorial influences on patient adverse outcomes in ED settings through the design and development of the Integrated Model for Patient Acuity, Care, and Triage Efficiency (IMPACT). Using ED presentation data, statistical inference, and analytics are employed to assess a comprehensive range of variables, including demographic characteristics, Triage Scores (TS), Complexity Scores (CS), Waiting Time (WT), and LoS. This study extends outcome evaluation beyond immediate events to include mortality assessments at 10- and 28-day intervals post-discharge. While adverse outcomes can encompass a range of clinical events, this study focuses specifically on mortality due to its objective measurability, clinical significance, and data reliability. Mortality provides a robust endpoint that reflects the cumulative impact of upstream factors such as delayed care, complexity, and acuity, making it an appropriate proxy for overall adverse outcomes in the context of ED performance evaluation. In addition, this study aims to generate an adverse outcome risk score to assist in clinical decision-making, optimise care delivery, and enhance operational efficiency within ED settings.

## Methods

The study aims to statistically analyse the impact of interconnected factors on patient adverse outcomes in the ED to develop IMPACT, a risk scoring system designed to support clinical decision-making and improve operational efficiency. Besides securing ethical approval, we followed the Transparent Reporting of a multivariable prediction model for Individual Prognosis Or Diagnosis (TRIPOD) guidelines [18, 19] to ensure methodological rigour and transparent reporting. This provides a comprehensive framework for developing and validating prediction models in healthcare research. The following sub-sections are structured according to the TRIPOD checklist to ensure clarity, reproducibility, and alignment with best practices in prediction model research.

### Data Source

We used Waikato Hospital data, covering ED presentations from January 2016 to August 2024. This dataset, extracted from the National Minimum Dataset (NMDS) [20] and National Non-Admitted Patient Collection (NNPAC) [21], national administrative datasets maintained for hospital and outpatient records, ensuring high-quality, standardised data. The dataset encompasses patient demographics (e.g., age, sex, and ethnicity), triage assessments and temporal data (e.g., arrival and discharge times), Operational metrics (e.g., WT and LoS), alongside clinical indicators (e.g., CSs, diagnostic and procedure codes). The dataset also includes comprehensive outcome measures, capturing mortality events within the ED, during inpatient care, and at specific intervals post-discharge. This rich dataset provides a robust foundation for analysing the complex interactions among factors affecting patient outcomes in emergency care settings.

### Study Population

We included all patients presenting to the Waikato Hospital ED during the specified period to create a complete and unbiased representation of the ED population. No exclusion criteria were applied to maximise generalisability. This diverse cohort allows for a comprehensive analysis of factors affecting patient outcomes across different demographic subgroups. Through this approach, we captured the full range of patient characteristics and clinical scenarios encountered in emergency care. To maintain patient confidentiality and comply with ethical standards, all data were de-identified, with strict protocols in place to ensure data security and privacy throughout the research process.

### Outcome

The primary outcome measures include the risk of mortality, defined as an adverse outcome occurring within the ED, within 10-days post-discharge, and within 28-days post-discharge. These time points were selected to capture both immediate and delayed mortality outcomes, providing a comprehensive assessment of patient survival following ED presentation. These outcomes served as target variables for the development and validation of the IMPACT scoring system, ensuring the model accounts for short-term and medium-term mortality risks. Statistical analysis evaluates the combined effect of key patient and operational factors on these outcomes, supporting the identification of critical predictors and the construction of a clinically applicable risk scoring system.

### Predictors

The study examines key predictors, including continuous variables such as age, WT, LoS, and CSs, alongside categorical variables including ethnicity, sex, and TS. These variables were selected based on their established importance in determining emergency care outcomes. Age, WT, LoS, and CSs represent important indicators of patient condition, care delays, and clinical complexity, all of which are associated with adverse outcomes [22–25]. Ethnicity and gender were included to account for known demographic disparities in healthcare access and outcomes [26, 27]. TSs were incorporated as an immediate clinical severity assessment recorded at presentation [28]. The CSs were normalised using Z-score normalisation to ensure comparability across different scales and stabilise the effect estimates in regression models [29]. Categorical variables were aggregated into clinically relevant categories to enhance statistical power and interpretability.

### Sample Size

The dataset initially contained 724,830 records, representing a substantial collection of ED presentations over the specified study period. No formal sample size calculation was done because the study is retrospective and based on available data. After preprocessing and cleaning, including imputation of missing values, aggregation of categories, normalisation of CSs, and removal of unrealistic values, the dataset was filtered to 593,673 records to improve consistency.

### Missing Data

As ED presentation data and outcome reporting are routinely captured in New Zealand hospital records, adverse outcomes were assumed to be complete. Missing values for WT and LoS were imputed with zero, on the basis that missing values for these variables predominantly occurred in records where the administrative pathway was incomplete or the patient self-discharged, resulting in negligible actual waiting or care duration. Missing TS values were assigned the lowest urgency category (TS = 5), reflecting the clinical assumption that the absence of a formal triage classification is most consistent with presentations not requiring urgent prioritisation. These are acknowledged as strong assumptions; the potential impact of alternative imputation strategies is discussed in the Limitations. Records with missing critical predictor variables were excluded from the final analysis. Data cleaning also involved removing implausible entries, such as negative or extreme WTs, which are treated as outliers. Following these steps, a complete case analysis was used, based on the assumption that the missing data were missing at random, supported by the observation that included and excluded cases had similar baseline characteristics.

### Data Analysis

To understand the dataset, identify key patterns, and uncover relationships between patient characteristics and adverse outcomes in the ED, a detailed data analysis was performed, which is detailed in the results section. This process was crucial for detecting trends, outliers, and potential data inconsistencies that could impact the accuracy of the results. The descriptive statistics and visualisations support data quality assessment, highlight underlying patterns, and inform subsequent statistical modelling.

### Statistical Analysis

The analysis employed a two-step modelling approach. A Generalised Linear Model (GLM) identified significant predictors of mortality as an adverse outcome, providing initial insights into the relationships between patient characteristics and outcome risk [30]. GLM was selected for its ability to provide initial insights into the strength and direction of associations between patient characteristics and adverse outcome risk, offering a transparent and interpretable starting point for model development. The second step implemented regularised logistic regression methods, including LASSO, Ridge and ElasticNet [31–33] to address potential multicollinearity among predictors and enhance model generalisability. The LASSO approach performs variable selection by shrinking some coefficients to zero, effectively removing less important predictors from the model [32]. Ridge regression penalises large coefficients to prevent overfitting, particularly when predictors are highly correlated [33]. The ElasticNet combines both techniques, applying LASSO’s feature selection and Ridge’s shrinkage simultaneously, which helps handle multicollinearity and improves model stability and predictive performance [31]. This hybrid modelling strategy was deliberately selected to balance predictive accuracy with interpretability. Unlike complex black-box Artificial Intelligence (AI) methods, the GLM and ElasticNet approaches allow transparent estimation of effect sizes and directionality for each predictor, ensuring that clinical stakeholders can trace model behaviour and understand its rationale for risk estimation.

To evaluate out-of-sample predictive performance and guard against overfitting, the dataset was randomly partitioned into 80% training and 20% testing subsets prior to any model development. All reported performance metrics, including AUROC, precision, recall, F1-score, and accuracy, were computed exclusively on the held-out test subset. No information from the test set was used at any stage of model fitting or predictor selection. These metrics were selected to provide a comprehensive assessment of the models’ discriminative ability and robustness [34]. AUROC was used to evaluate the models’ ability to distinguish between patients at different adverse outcome risks across a range of threshold values, which is critical for risk stratification in clinical settings. Precision, recall, and F1-score were included to ensure balanced evaluation of model performance, particularly given the low event rate for the outcomes. Accuracy was reported to provide an overall measure of correct classifications but was interpreted alongside the other metrics to account for class imbalance.

An adverse outcome risk scoring system was developed based on ElasticNet logistic regression coefficients due to its ability to simultaneously perform variable selection and prevent overfitting in high-dimensional settings [35]. The system calculates individual patient risk through a linear combination of predictors as it is shown in Eq. (1).

1 $$\:M={\beta\:}_{0}+{\beta\:}_{1}\left(Age\right)+{\beta\:}_{2}\left(WT\right)+{\beta\:}_{4}\left(LoS\right)+\dots\:+{\beta\:}_{n}\left({Predictor}_{n}\right)$$

Where *M* represents the linear predictor, *β₀* is the intercept, *β₁*, to *βₙ* coefficients for each predictor, and the predictor variables represent patient-specific values. The coefficients were derived from the ElasticNet model, which balances feature selection and regularisation to provide stable and interpretable estimates of predictor importance.

To convert the linear predictor (*M*) into a probability of adverse outcome, the logistic function was applied which is formulated in Eq. (2).

2 $$\:P=\frac{{e}^{M}}{1+{e}^{M}}$$

**Fig. 1:**
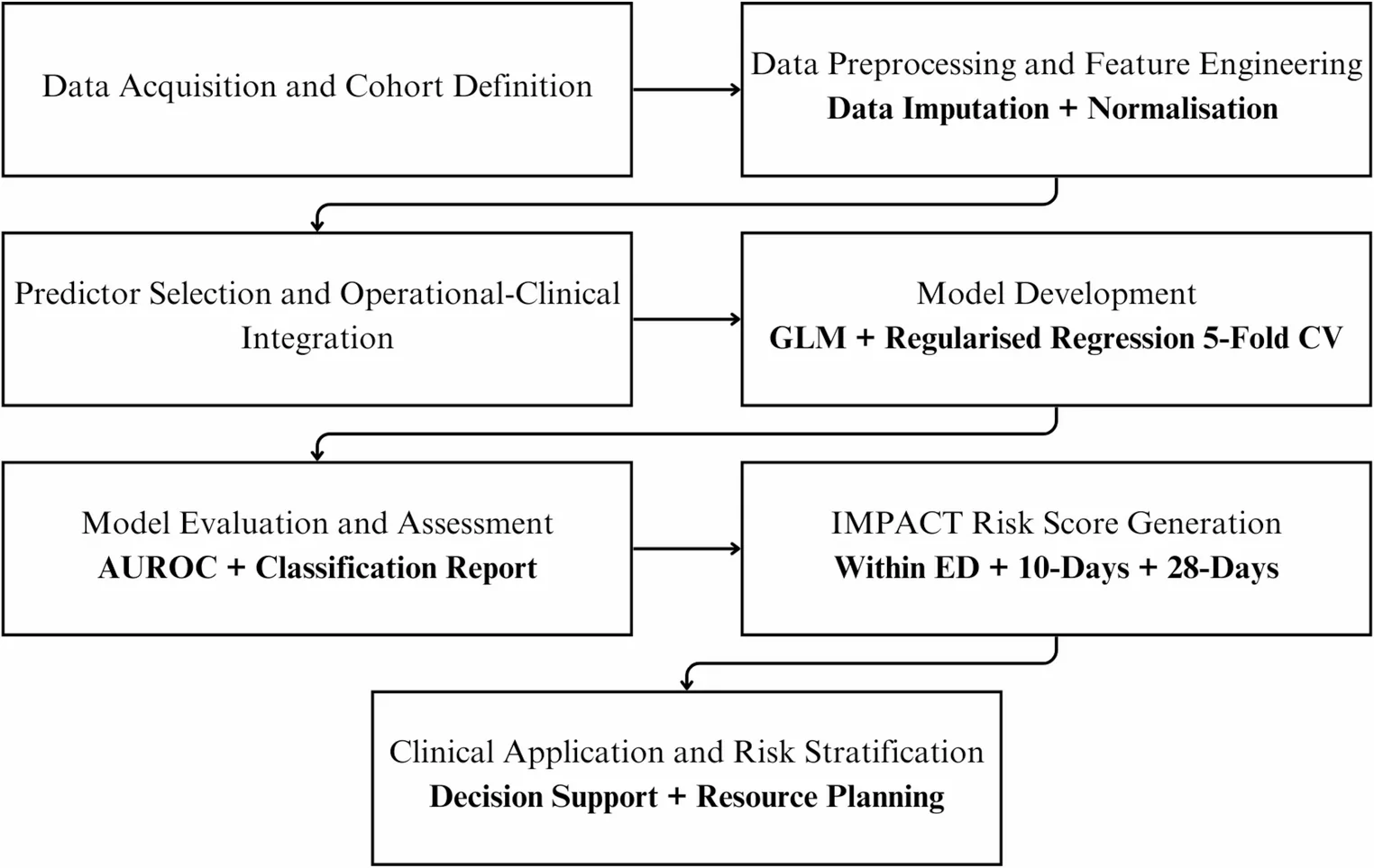
Development pathway of the IMPACT risk score

This transformation ensures risk scores range from 0 to 1, with higher values indicating greater probability of adverse outcome risk [36–38]. The resulting probability was designated as the IMPACT risk score. This quantitative measure of individual patient risk can be interpreted and applied in clinical practice, enabling healthcare providers to identify high-risk patients and implement appropriate interventions to improve outcomes. The predictive performance of the IMPACT score was subsequently evaluated across ED mortality, 10-days mortality, and 28-day mortality outcomes. The development process of IMPACT risk score, including data acquisition, model construction, score generation, and clinical application, is summarised in Fig. 1.

## Results

We performed data preprocessing and cleaning using Python 3.13.1, Pandas, NumPy, sci-kit-learn, and seaborn. At this stage, ethnic groups were standardised for analysis by aggregating granular categories into broader, interpretable groups. For example, Pacific Island ethnicities were grouped under “Pacifica,” while unknown or unclassifiable responses were combined into “Other/Unspecified.” Mortality indicators, initially distributed across multiple columns under various conditions, were consolidated into derived binary variables representing overall mortality. Specific indicators, such as mortality in the ED and mortality within 10- and 28-day post-discharge, were retained and analysed separately.

To address missing values and inconsistencies, numeric columns such as WT, LoS, and TS were processed according to clinical practices. Missing WT and LoS values typically occurred when patients were discharged, deceased, or left the ED before formal processing; these were imputed with zero to reflect the absence of measurable waiting or stay time. For TS, missing entries indicated unrecorded scores, which by default corresponded to the lowest urgency level; these were replaced accordingly. WTs originally recorded in minutes were converted to hours. Negative and extremely high values were excluded to enhance interpretability and reduce their influence on the analysis.

CSs, were recorded using two scaling systems: Patient Clinical Complexity Level (PCCL) until 2023, and Episode Clinical Complexity Score (ECCS) [39] thereafter, resulting in inconsistency. To enable direct comparisons, all CS values were normalised using Z-score normalisation, centring around zero with unit variance, preserving distributional properties [40]. Row missing complexity scores were marked as zero by default, and a unified complexity metric was created.

### Exploratory Data Analysis

The cleaned and wrangled dataset contained 593,673 records. Table 1 presents descriptive statistics for continuous variables and the distribution of categorical variables, outlining characteristics used in the analysis. The mean patient age was 40.73 years and a range of 0 to 121 years. The mean hospital stay for ED patients was 5.16 h, while the average WT in the ED was 1.62 h. After Z-score normalisation, clinical scores (CS) ranged from − 0.99 to 0.85 with a mean of -0.01.

In the demographic distribution, NZ European constituted the largest ethnic group at 50.5%, followed by Māori (29.1%), European (7.6%), Pacifica (3.5%), Asian (3.4%), and Indian (3.3%). Other represented groups were Middle Eastern, Latin American, and African (MELAA) at 1.7%, and Other/Unspecified at 0.9%. The sex distribution was relatively balanced, with 51.1% males and 48.9% females.

In terms of triage distribution, the majority of patients were categorized as Score 3 (53.8%), followed by Score 4 (24.9%) and Score 2 (17.7%). Less frequent were Score 5 (3.2%) and Score 1 (0.3%), indicating that most patients presented with moderate levels of urgency rather than the most critical or least urgent categories.

Regarding mortality, the vast majority of patients survived across all time frames. ED mortality accounted for 0.7% of cases, while 10-days and 28-days mortality rates were 0.4% and 0.5%, respectively. These relatively low mortality rates highlight the overall resilience of the study population, while underscoring the importance of identifying subtle predictors of adverse outcomes.

**Table 1:** Descriptive analysis

Analysis Dataset (*n* = 593,673)		
	Continuous Variables (Mean)	
	Age	40.73
	Waiting Time	1.62
	Length of stay	5.16
	Complexity Score	-0.01
	Categorical Variables (%)	
Sex	Male	303,237 (51.1)
	Female	290,436 (48.9)
Triage Score	Score 1	1886 (0.3)
	Score 2	105,253 (17.7)
	Score 3	319,317 (53.8)
	Score 4	147,957 (24.9)
	Score 5	19,260 (3.2)
Ethnicity	NZ European	299,525 (50.5)
	Māori	172,992 (29.1)
	European	45,187 (7.6)
	Pacifica	20,925 (3.5)
	Asian	20,453 (3.4)
	Indian	19,575 (3.3)
	MELAA	9856 (1.7)
	Other/Unspecified	5160 (0.9)
Mortality	ED Mortality	3862 (0.7)
	10-Days Mortality	2466 (0.4)
	28-Days Mortality	3251 (0.5)

**Fig. 2:**
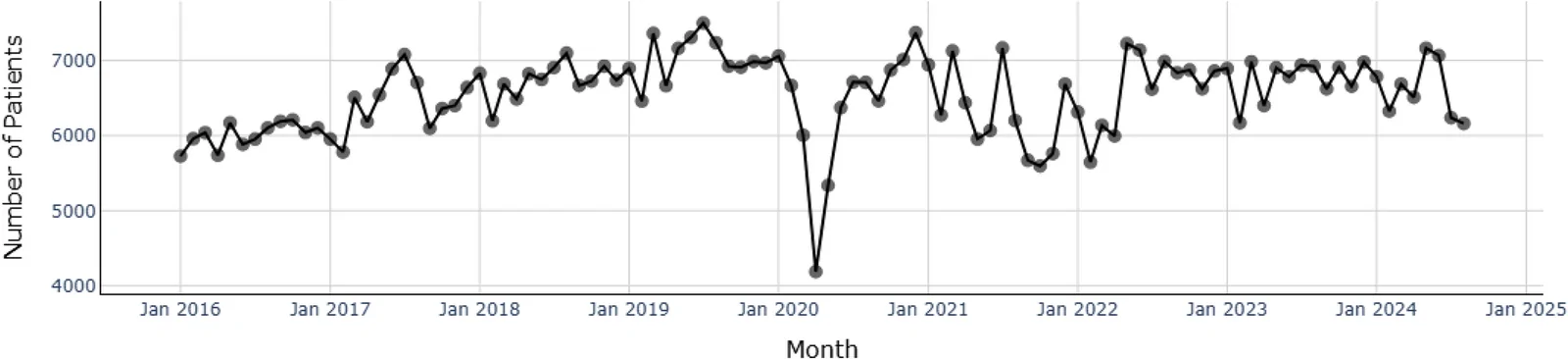
Annual trends in ED patient arrivals

Following the descriptive analysis, the study explored trends in patient arrivals over time to better understand temporal patterns in ED utilisation for operational purposes, such as resource planning, staffing, and improving service delivery. Figure 2 illustrates the monthly number of patients arriving at the ED between January 2016 and January 2025. This figure shows a steady month-on-month variation in patient arrivals, with an upward trend from 2016 to 2019. The arrival pattern fell significantly at the beginning of 2020 due to the COVID-19 pandemic, likely reflecting a reduction in in-person visits to the ED [41] From this decline, the arrivals slowly regained their pace and, after a recovery period, levelled off, with some stability but a little lower from late 2020 onwards. The time series exhibits seasonal variations, which can be interpreted as cyclic demand related to factors such as winter illnesses or other seasonal healthcare needs.

**Fig. 3:**
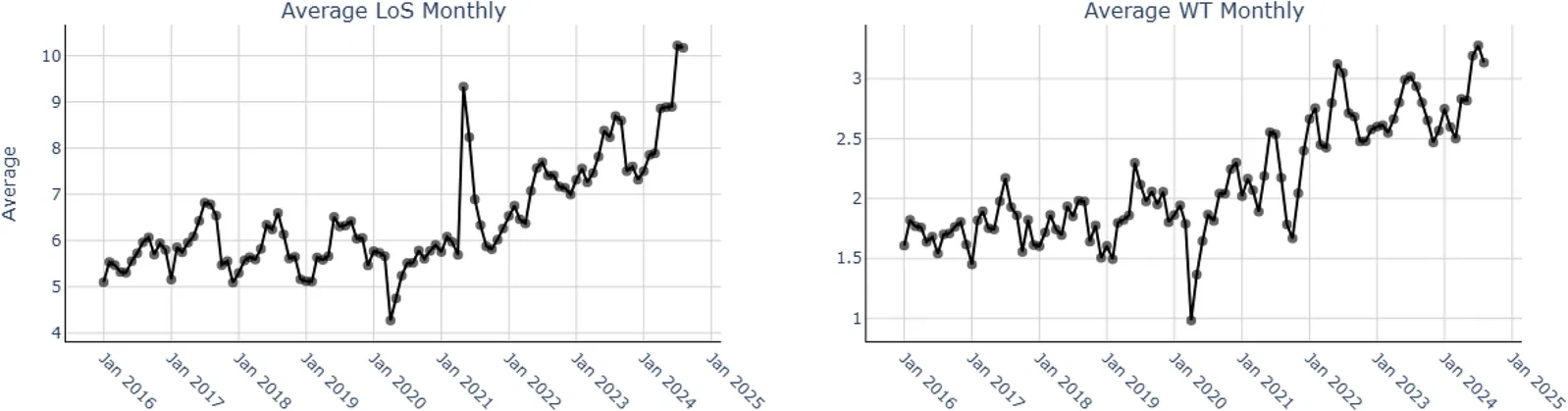
Monthly trends for average LoS and average WT in the ED

Building on the trends for patient arrivals, Fig. 3 drills deeper into the operational metrics of the ED concerning average LoS and WT over the same period. The average LoS follows an upward trend with wide spikes during 2020–2021 and then shoots steeply toward the end of 2024, indicating either an increase in the complexity of the cases or systemic inefficiency. The average WT follows a similar upward trend, except for the drop at the beginning of 2020, due to reduced patient arrivals in the wake of the COVID-19 pandemic. This suggests that pressure on ED operations has been gradually increasing and justifies the need for further investigation to understand better and address the underlying factors.

**Fig. 4:**
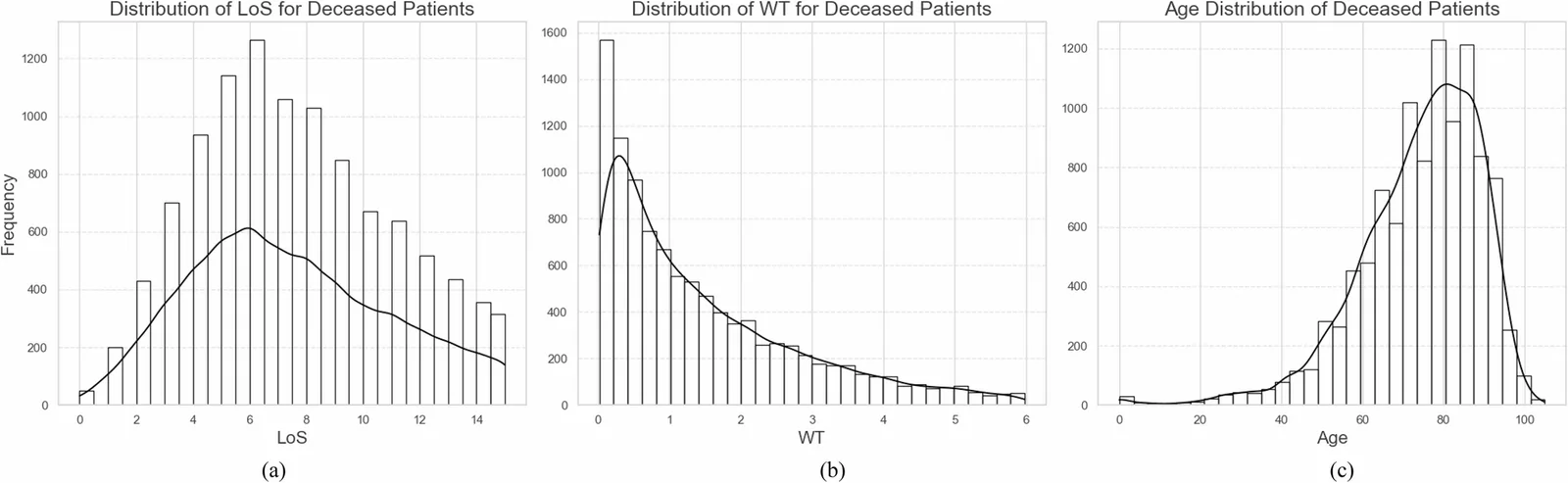
Distribution of LoS (**a**), WT (**b**), and age (**c**) among deceased patients

Figure 4 extends the trends observed in Fig. 3. It provides LoS, WT, and age distributions for deceased patients. This figure presents critical information about the nature of patients who died.

Figure 4 (a) presents the LoS distribution for deceased patients. The distribution is unimodal, with most patients stayed in the ED between 4 and 8 h, peaking around 6 h. Extended LoS may indicate higher patient complexity or delays in care, both potentially linked to increased risk of adverse outcomes. These findings emphasise the the importance of reducing prolonged LOS to potentially improve survival rates.

Figure 4 (b) shows the WT distribution for deceased patients, which is heavily skewed, with most patients experiencing a WT of less than 1 h. This suggest that ED prioritises immediate care for critically ill patients. However, a subset of the deceased patients experienced longer WT, which may negatively affect their outcomes. This emphasises the need to ensure minimum delay, particularly in cases related to life-threatening conditions, to improve overall quality of care.

**Fig. 5:**
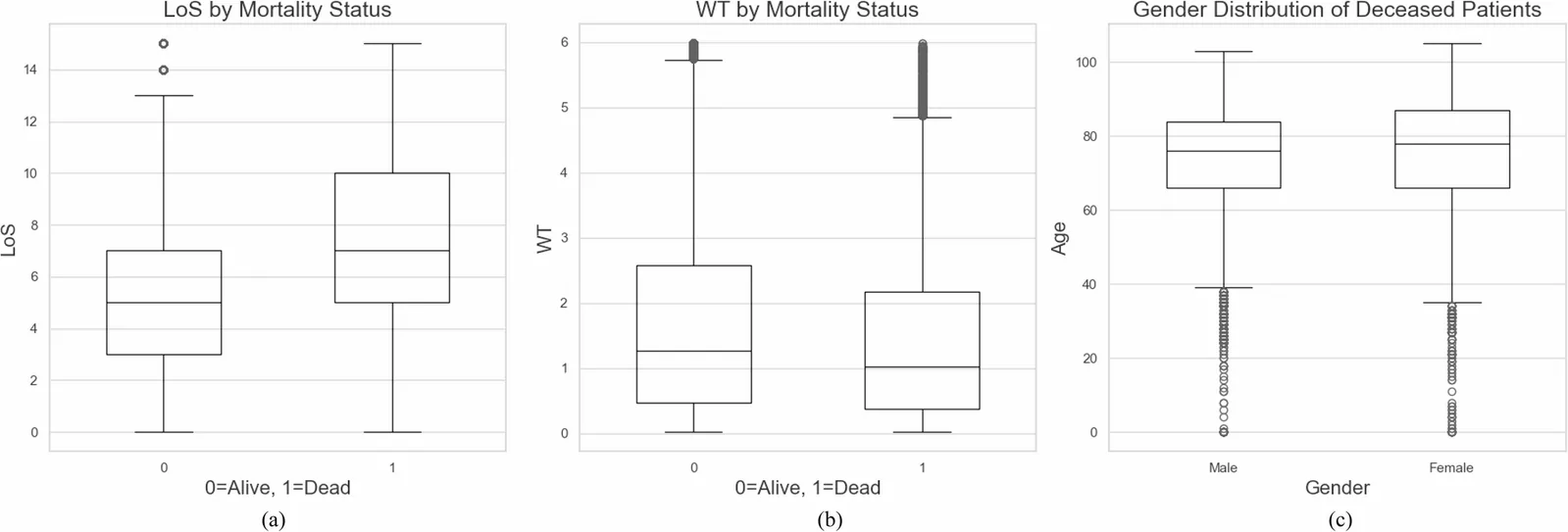
Box plots comparing LoS, WT, and gender distribution by mortality

Figure 4 (c) considers the age distribution of the deceased patients. It follows that adverse outcome is highly concentrated in the older age groups. Most of the deaths happened in patients aged between 70 and 90 years, reflecting the fact that older patients are more vulnerable because of frailty, comorbidities, and reduced physiological reserves. On the other hand, mortalities were very few among the younger patients, in line with the relative resilience of this demographic. Figure 4 indicates that shorter WT shows the responsiveness of EDs for critical cases, while longer LoS indicates potential bottlenecks in handling complex cases. Furthermore, strong age-related trends underscore the need for tailored approaches to improve outcomes among older patients, who account for the majority of mortalities. Figure 5 builds on these insights by further exploring the relationships between LoS, WT, age, and gender in relation to mortality status.

Figure 5(a) compares the LoS for patients grouped by mortality status: 0 (alive) and 1 (deceased). The median LoS for the deceased patients was considerably higher, with the Interquartile Range (IQR) and maximum values extending well beyond those of survivors. This suggests that patients who eventually died stayed longer in the ED, likely due to the complexity of their conditions or clinical deterioration. Figure 5 (b) presents the WT by mortality status. The median WT for both groups was nearly similar, reflecting that deceased patients were subjected to prolonged delays before receiving care. These results underscore that although most critically ill patients were prioritised, a subset of deceased patients suffered substantial delays that likely contributed to their poor outcomes. Figure 5 (c) plots the distribution of the age of deceased patients by gender. The plot shows that males and females exhibit similar age distributions. No large differences in medians or IQRs, but the presence of deceased among younger patients underlines the variability in adverse outcomes across different age and gender groups.

**Fig. 6:**
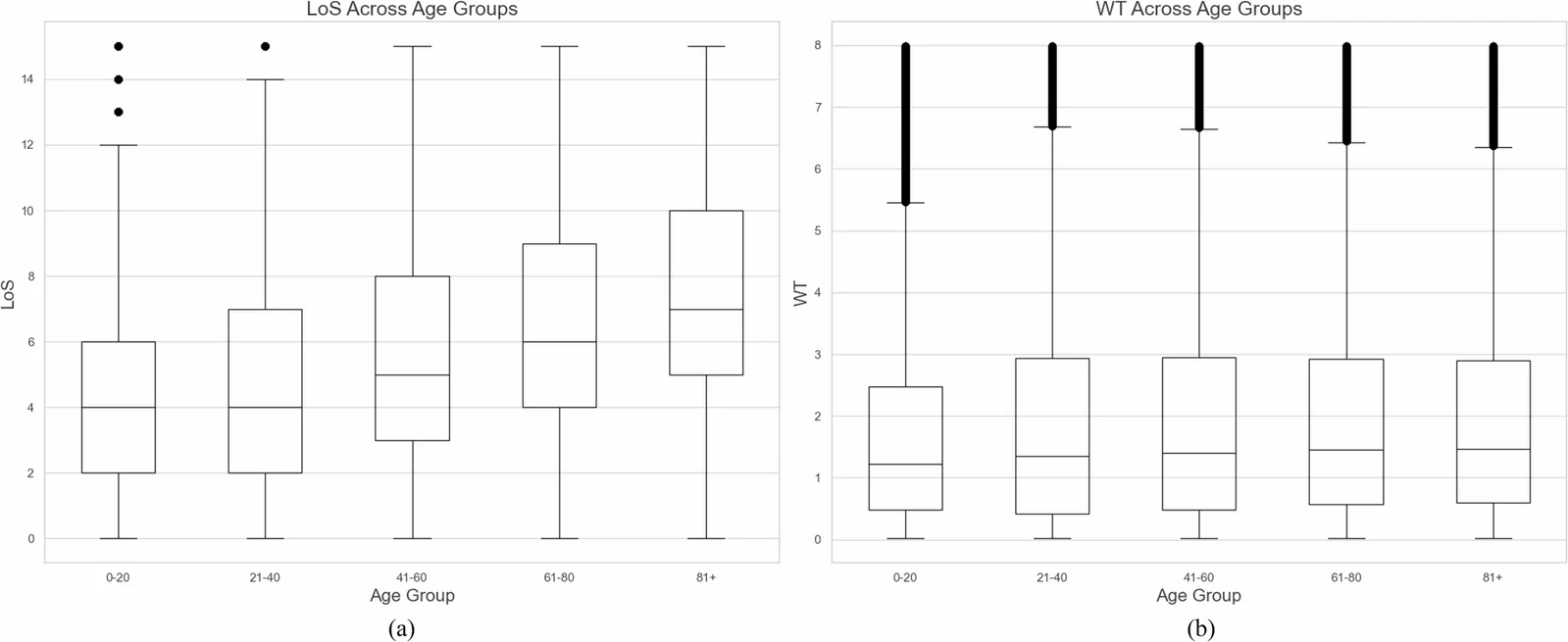
Box plots of LoS (**a**) and WT (**b**) across age groups

Given the emphasis on LoS and WT as key drivers of patient outcomes, the analysis was extended to explore how these variables vary with age. Figure 6 illustrates this comparison by showing the distribution of LoS (a) and WT (b) across five age categories.

The trend in Fig. 6 shows the median of LoS rises with age. Younger age groups, for example, 0–20 and 21–40 years, have shorter LoS, meaning that their stay durations are less variable. Older patients, especially those aged 61–80 and 81+, have a median LoS representing longer and more variable stays, mainly due to higher complexity and greater healthcare need. The right panel plots the distribution of WT by age. Here, again, median WT is relatively constant, but the IQRs are wider at older group ages than 0–20. Such patterns imply that most patients get timely care irrespective of age, but some older individuals receive significantly delayed care, which can exacerbate risks for adverse outcomes.

**Fig. 7:**
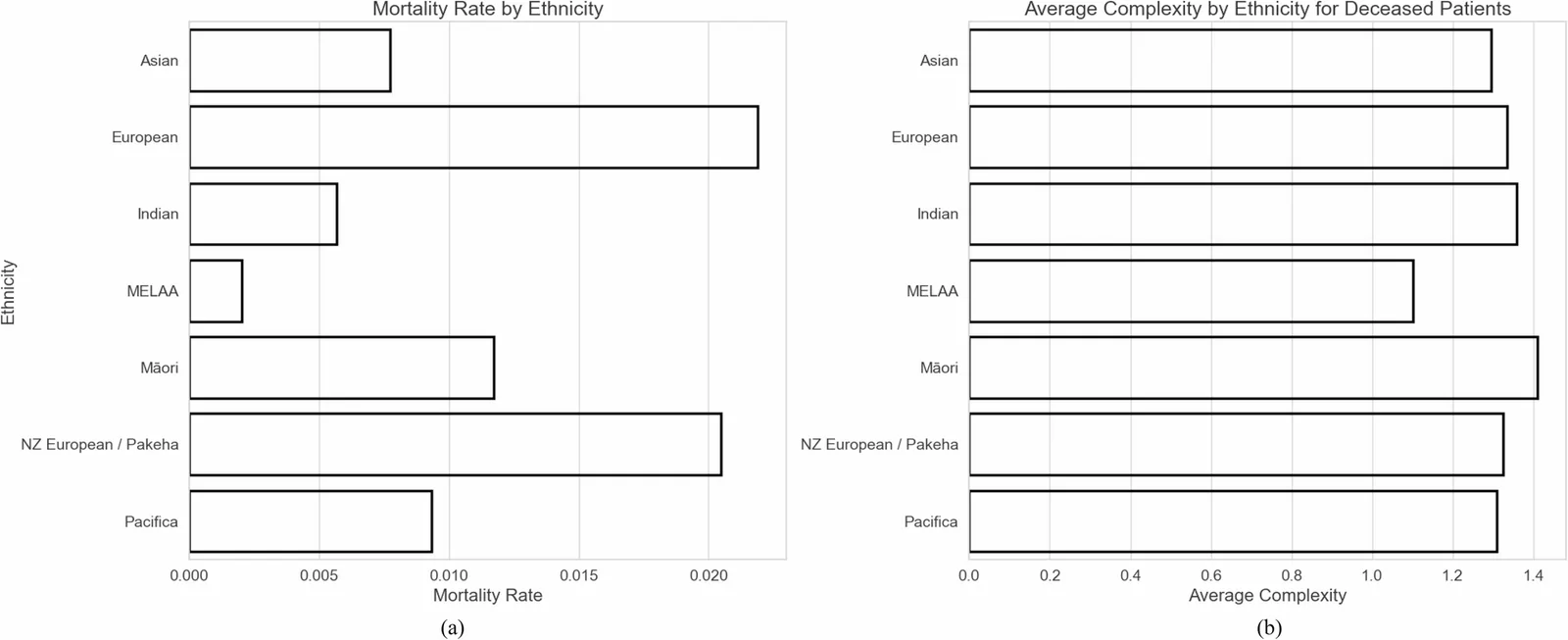
Mortality rates and average CSs for deceased patients

Continuing the analysis, Fig. 7 gives the mortality rates (a) and the average CS (b) for deceased patients grouped by ethnicity. Figure 7 (b) reveals notable disparities in mortality rates across ethnic groups. European patients exhibit the highest mortality rates, followed by NZ European, Pacifica and Māori populations. In contrast, Asians, MELAA, and Indians have rather low mortality rates. Māori patients provide the most significant proportion in ED and have moderately high mortality rates; special attention is called for regarding the issues in health that Māori are experiencing. Their higher mortality despite comparable triage profiles suggests structural factors, such as delayed access, differential follow-up, or systemic bias that may influence outcomes. Quantifying these gaps establishes a foundation for targeted equity monitoring within the IMPACT framework.

Figure 7(b) focuses on the average CSs for deceased patients, providing additional information on these mortality trends. Māori and Indian populations have the highest average CSs, which means that patients from these groups often come into the hospital with more advanced or severe conditions, probably due to a delay in getting care or a higher prevalence of comorbidities. Asian and MELAA populations have relatively lower mortality rates and report lower average clinical scores, thus perhaps linking clinical complexity to outcome.

### 3.2 Model Outcomes

A two-step modelling approach was employed, involving GLM for statistical inference and regularised logistic regression to improve performance while minimising overfitting and multicollinearity. Table 2 represents the result of the GLM for all three categories of adverse outcomes.

**Table 2:** The results of the GLM

		ED Mortality	10-Days Window	28-Days Window
Variables		Coefficients		
	Intercept	-1.01	-3.28	-3.86
Ethnicity				
	European	-0.25	0.05	0.08
	Indian	-0.46	-0.25	-0.28
	MELAA	-0.91	-0.46	-0.14
	Māori	0.0031	0.56	0.50
	NZ European	-0.15	0.31	0.26
	Other/Unspecified	0.22	0.82	0.98
	Pacifica	0.18	0.44	0.06
Sex				
	Male	0.04	0.13	0.18
	Age	0.05	0.06	0.06
	Waiting Time	-0.13	-0.06	-0.04
	Length of Stay	0.03	0.04	0.04
	Triage Score	-1.27	-0.56	-0.30
	Complexity Score	1.50	1.02	0.85

The results show negative intercepts across all models, indicating a low baseline likelihood of mortality when no predictors are considered. Ethnicity significantly influences mortality. Indian and MELAA groups consistently show negative coefficients, with MELAA having the strongest protective effect. In contrast, Māori and Pacifica exhibit increased odds of mortality. Māori show minimal impact on ED mortality but have elevated odds in the longer timeframes, suggesting potential disparities that warrant further investigation. Age is a predictor of increased mortality, with positive coefficients across all categories. Similarly, CSs are consistently associated with higher mortality chances, reflecting the severity of patients’ conditions. In contrast, higher TSs, indicating less severe cases, are linked to significantly reduced mortality probability.

Gender shows a mild impact, with males having slightly higher mortality odds than females across all categories. LoS has a marginally positive association with mortality, suggesting that extended hospitalisations may correlate with more severe health issues. However, WT exhibits a negative relationship with mortality, implying that longer waits are associated with lower odds of death. This counterintuitive finding may indicate unmeasured confounding factors influencing the relationship, such as differences in resource allocation or triage processes. Therefore, Logistic Regression is necessary here to model the binary nature of mortality outcomes effectively, capturing the relationship between predictors and the probability of mortality. Regularisation techniques, such as LASSO, Ridge, or ElasticNet, are crucial for addressing issues like multicollinearity among predictors and preventing overfitting, especially in complex models with multiple variables. Regularisation improves the model’s generalisability and ensures more reliable inferences.

The regularised logistic regression results in Table 3 confirm and refine the GLM findings while addressing overfitting and multicollinearity. Across all models and adverse outcome windows, higher CSs and age remain strong positive predictors of mortality. A longer LoS also consistently increases mortality odds. WT continues to show a negative association with mortality, but its magnitude is small and warrants further investigation to uncover potential confounding factors.

**Table 3:** Regularised Logistic Regression Coefficients (LASSO, Ridge, ElasticNet)

Variables		ED Mortality			10-Days Window			28-Days Window		
		LASSO	Ridge	EN	LASSO	Ridge	EN	LASSO	Ridge	EN
	Intercept	3.61	3.61	3.61	0.63	0.58	0.58	-0.40	-0.38	-0.40
	Age	1.49	1.49	1.49	1.60	1.60	1.60	1.61	1.60	1.61
	Wait Time	-0.19	-0.19	-0.19	-0.13	-0.13	-0.13	-0.09	-0.09	-0.09
	Length of Stay	1.15	1.15	1.14	1.19	1.17	1.21	1.26	1.18	1.26
	Complexity Score	1.74	1.74	1.74	1.15	1.15	1.15	0.93	0.93	0.93
Ethnicity										
	European	0.02	0.02	0.02	0.04	0.01	0.04	-0.02	-0.03	-0.02
	Indian	-0.24	-0.24	-0.24	-0.25	-0.25	-0.26	-0.40	-0.35	-0.40
	MELAA	-0.56	-0.56	-0.56	-0.44	-0.41	-0.44	-0.24	-0.21	-0.24
	Māori	0.22	0.22	0.22	0.57	0.54	0.57	0.40	0.38	0.40
	NZ European	0.09	0.09	0.09	0.31	0.28	0.31	0.16	0.14	0.16
	Other/Unspecified	0.49	0.49	0.49	0.82	0.77	0.82	0.82	0.74	0.82
	Pacifica	0.46	0.46	0.46	0.47	0.44	0.47	-0.06	-0.06	-0.06
Sex										
	Male	0.02	0.03	0.02	0.14	0.14	0.14	0.19	0.18	0.19
Triage Score										
	Score 2.0	-3.22	-3.22	-3.22	-0.87	-0.80	-0.82	0.09	0.08	0.09
	Score 3.0	-4.29	-4.29	-4.29	-1.11	-1.03	-1.06	0.06	0.06	0.06
	Score 4.0	-5.15	-5.15	-5.15	-1.98	-1.90	-1.93	-0.53	-0.54	-0.53
	Score 5.0	-5.42	-5.41	-5.41	-3.00	-2.80	-2.91	-0.87	-0.80	-0.87

Ethnicity remains a significant predictor. MELAA consistently shows reduced odds of mortality, while Pacifica and Unspecified groups display higher odds, particularly for the 10-days window. Māori demonstrate moderately elevated odds of mortality compared to other groups. The results also highlight sex differences, with males having slightly higher likelihood of mortality. To further understand the impact of triage on mortality, TSs were categorised into discrete groups. The results reveal a strong protective effect as TSs increase. Patients with scores of 4 or 5 (indicating lower urgency) are significantly less likely to die across all mortality windows, with progressively lower odds as the score increases.

**Fig. 8:**
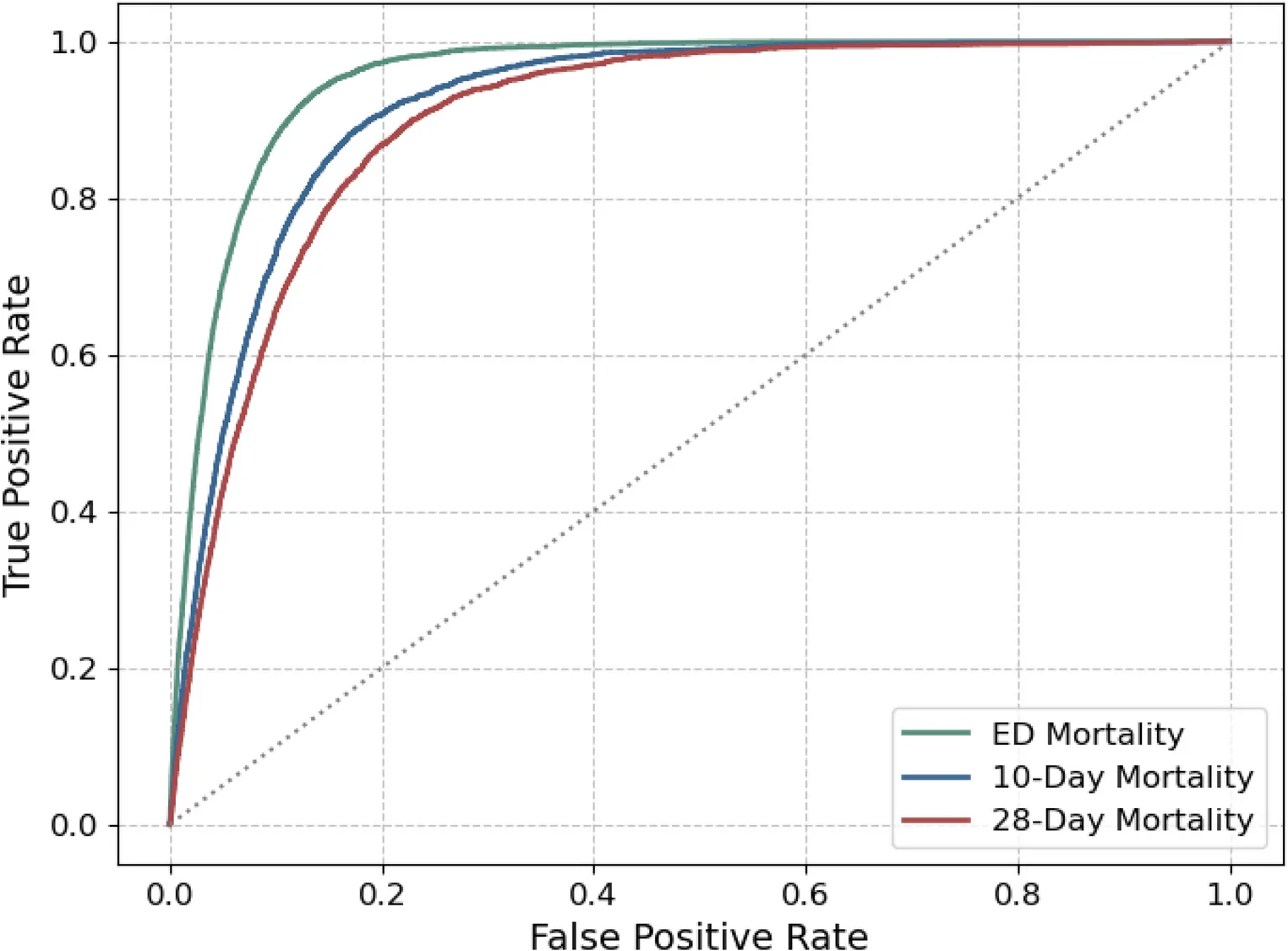
AUROC plots for the Elastic Net model for all mortality categories

Upon analysis, the coefficients derived from the LASSO, Ridge, and ElasticNet models exhibited considerable similarity across all adverse outcome prediction windows. Given that ElasticNet integrates the strengths of both LASSO’s variable selection and Ridge’s management of multicollinearity, it was selected to report the statistical results. The dataset was randomly split into 80% for model development and 20% for final evaluation. In addition, model development was performed on the training set using 5-fold cross-validation to optimise regularisation parameters and ensure robust coefficient estimation. This approach effectively balances bias and variance, providing stable and interpretable coefficients, which is particularly advantageous in datasets with correlated predictors. The combined regularisation of ElasticNet ensures robustness in identifying key predictors such as age, complexity score, and ethnicity, making it well-suited to capturing nuanced relationships within the data. Figure 8 presents the AUROC values for the ElasticNet model, demonstrating its predictive performance across the different mortality categories.

The classification report for the ElasticNet models, summarised in Table 4, further reinforces the model’s predictive reliability across different adverse outcome timeframes. The model demonstrates strong classification performance for ED Mortality, achieving an overall accuracy of 0.90, with a high precision-recall balance for both alive (precision: 0.91, recall: 0.88) and deceased (precision: 0.89, recall: 0.92) cases, ensuring robust differentiation between survival outcomes. For the 10-Days Mortality model, the accuracy remains high at 0.86, with similar recall values (alive: 0.83, deceased: 0.88), reflecting its ability to capture short-term mortality risks with reliable sensitivity and specificity. The 28-Days Mortality model maintains a competitive performance with an accuracy of 0.83, where a slightly lower recall for alive cases (0.80) suggests a marginal trade-off in sensitivity for extended adverse outcome predictions. Across all models, the f1-scores remain consistently strong, indicating a well-balanced trade-off between precision and recall. These findings align with the AUROC values presented in Fig. 8, affirming the ElasticNet model’s capability to generalise effectively across different adverse outcome periods while preserving both interpretability and predictive robustness.

**Table 4:** Classification report for Elastic-Net models

Model	Status	Precision	Recall	f1-Score	Accuracy	AUROC (95% CI)
Ed-Mortality	Alive	0.91	0.88	0.89	0.90	0.953 (0.943–0.964)
	Dead	0.89	0.92	0.90		
10-Days Window	Alive	0.87	0.83	0.85	0.86	0.918 (0.902–0.935)
	Dead	0.84	0.88	0.86		
28-Days Window	Alive	0.86	0.80	0.83	0.83	0.900 (0.884–0.916)
	Dead	0.81	0.87	0.84		

### 3.3 IMPACT Risk Scoring

The IMPACT risk scoring system provides a structured method to calculate patient-specific risk scores based on predictors identified through the ElasticNet logistic regression model. For example, the risk score for ED mortality is calculated by combining relevant predictors, such as age, waiting time, length of stay, complexity score, ethnicity, sex, and triage score, using regression coefficients derived specifically for this model. The resulting linear predictor is then converted into a risk probability using the logistic function. As an example, Eq. (3) shows how patients’ risk of ED Mortality can be calculated.

3 $$\:\begin{array}{c}{M}_{ED}=3.61+1.49\left(Age\right)+\left(-0.19\right)\left(WT\right)+1.14\left(LoS\right)+\\\:1.74\left(CS\right)+0.02\left(Sex\right)+\left(-3.22\right)\left(TS2\right)+\left(-4.29\right)\left(TS3\right)\\\:+\left(-5.15\right)\left(TS4\right)+\left(-5.41\right)\left(TS5\right)+0.02\left(European\right)\\\:+\left(-5.15\right)\left(TS4\right)+\left(-5.41\right)\left(TS5\right)+0.02\left(European\right)+\\\:(-0.24)\left(Indian\right)+(-0.56)\left(MELAA\right)+0.22\left(Māori\right)+\\\:0.09\left(NZ\:Europ\right)+0.49\left(Other\right)+0.46\left(Pacifica\right)\end{array}$$

The resulting linear predictor is then converted into a risk probability using the logistic function as it is illustrated in Eq. (4)

4 $$\:{P}_{ED}=\frac{{e}^{{M}_{ED}}}{1+{e}^{{M}_{ED}}}$$

Similar risk-scoring equations can be constructed for predicting mortality within the 10-days and 28-days timeframes post-ED visit, using their respective model coefficients. Each equation generates a probability score ranging from 0 (lowest risk) to 1 (highest risk). Once calculated, these risk scores can be integrated directly into patients’ records, ensuring that comprehensive risk assessment data is readily available and continuously accessible within clinical systems.

## Discussion

Recognising the complex and dynamic nature of ED environments, where patient outcomes are influenced by a range of interrelated clinical, operational, and demographic factors, this research sought to move beyond the limitations of prior studies that predominantly examined individual risk factors in isolation. By systematically incorporating key predictors into a unified, analytically robust framework, IMPACT was designed to capture the synergistic interplay among these variables. This approach offers a more comprehensive and accurate assessment of patient risk, ultimately supporting improved clinical decision-making, resource allocation, and operational efficiency within emergency care settings. In addition, the interpretability of the IMPACT framework strengthens its clinical applicability. By combining a transparent logistic regression with ElasticNet regularisation, the model maintains clarity in how each variable contributes to patient risk while ensuring robust predictive accuracy.

The analysis revealed several critical insights into the multifactorial determinants of ED patient outcomes, particularly regarding operational metrics, clinical factors, and demographic disparities. One of the most notable findings was the negative association between WT and adverse outcome, contrasting with earlier studies that reported longer WT as a risk factor for increased mortality [23]. In this study, patients who waited longer demonstrated slightly lower odds of adverse outcome. This association should not be interpreted causally. Rather, it is consistent with triage-induced prioritisation effects. Patients with the highest acuity are triaged at lower TS scores, seen immediately, and are also at the greatest intrinsic risk of mortality. Consequently, shorter WT is a marker of clinical urgency, not of poorer care. WT therefore functions in this model as an operational correlate of acuity, not an independent protective factor. This system prioritises critically ill patients for immediate care, while less critical patients experience longer WTs. Consequently, shorter WT often reflects condition severity rather than care quality, indicating the need for urgent intervention. The discrepancy between our findings and previous research likely stems from differences in study settings, patient populations, and healthcare system structures, emphasising that WT alone cannot serve as a reliable indicator of care quality without considering contextual factors such as triage decisions, resource availability, and patient acuity. It is further noted that TS is included as an independent predictor within the IMPACT model, providing partial statistical control for the acuity confounding between WT and outcome. A formal WT × TS interaction term would address this confounding more rigorously and is identified as a priority for future model refinement.

Conversely, the positive association between LoS and adverse outcome was consistent across all timeframes. Patients who remained in the ED longer consistently exhibited higher risks, underscoring the impact of delays within the emergency care pathway. This relationship likely reflects several contributing factors, including delayed access to definitive care, complications arising from prolonged hospitalisation, and the greater clinical complexity of patients requiring extended ED management. These findings are consistent with prior research by Jones et al. [9] and Wessman et al. [25], reinforcing the established link between prolonged LoS and adverse outcomes. Importantly, the results highlight that systemic inefficiencies, such as bottlenecks in patient flow, delayed inpatient admissions, and constrained resource availability, are not merely operational challenges but directly translate into increased adverse outcome risks. Addressing LoS-related inefficiencies is therefore essential for both optimising ED operations and improving patient survival. These insights further illustrate how an interpretable analytical model can uncover operational drivers of mortality, enabling clinicians and managers to act on transparent, evidence-based findings rather than unclear statistical outputs.

Clinical complexity and demographic factors played crucial roles. Higher CS and advanced age were strong predictors of increased adverse outcome, reflecting the vulnerability of older patients due to frailty, comorbidities, and reduced physiological reserves. The protective effect of higher TS (indicating lower urgency) validated the effectiveness of triage systems in correctly identifying and prioritising patients based on clinical urgency, demonstrating the importance of accurate triage in improving ED efficiency and patient safety. While triage remains central to patient prioritisation, the IMPACT framework extends its clinical utility by quantifying the probability of adverse outcomes rather than assigning categorical urgency levels. As triage score is one of the model’s core predictors, IMPACT complements and strengthens current triage systems by translating patient and operational data into continuous risk estimates. Following prospective validation, this framework could enable clinicians to identify high-risk patients earlier and make more informed decisions about escalation and resource allocation.

Demographic disparities were evident, particularly concerning ethnicity. Māori and Pacifica populations exhibited higher mortality rates compared to other groups, while MELAA and Indian populations showed lower rates. These findings align with [17], which documented higher mortality and re-presentation rates among Māori compared to non-Māori populations in New Zealand. The persistence of these disparities, despite national health initiatives, underscores the complexity of addressing health inequities and the need for systemic changes in healthcare delivery, resource allocation, and cultural competence. These findings highlight the importance of adopting an integrative, multifactorial approach to adverse outcome risk prediction in ED settings, as initially proposed. In practical terms, the IMPACT framework can be used to flag disproportionate mortality risks among Māori and Pacifica patients during ED presentations, prompting early clinical review or escalation of care. At a policy level, aggregated IMPACT data can inform equity-focused performance indicators, guide targeted resource allocation across high-need regions, and support the design of culturally responsive clinical pathways and workforce development programmes aimed at reducing these disparities.

The study’s findings have substantial implications for ED policy development, operational planning and clinical practice. IMPACT is designed to integrate multifactorial risk factors into a unified scoring model for retrospective risk stratification and service evaluation. In future work, it offers the potential to inform ED workflow improvements through prospective risk assessment. When embedded within electronic health record systems, IMPACT could enable continuous, automated monitoring of incoming ED patients, identifying individuals at heightened risk through dynamic clinical alerts. This early identification facilitates timely clinical interventions, targeted diagnostic testing, and prioritised care pathways, thereby improving patient outcomes. Moreover, by stratifying patients based on objective risk metrics, the system can support more efficient resource allocation, ensuring that critical resources, such as intensive care beds, specialist consultations, and inpatient admissions, are prioritised for those who would benefit most [42].

Model calibration, the correspondence between predicted probabilities and observed event rates, is an important dimension of predictive model performance that complements discriminative accuracy. Formal calibration assessment has not been conducted in the present study and is acknowledged as a limitation. However, the use of ElasticNet regularisation inherently dampens coefficient magnitude and reduces the risk of overfitting, which generally supports well-calibrated probability estimates. The high AUROCs observed are consistent with the inclusion of strongly discriminative predictors, the discriminative value of which has been reported in comparable ED mortality models. Formal prospective calibration assessment will be required prior to any clinical deployment of the IMPACT score.

Given the dynamic and unpredictable nature of ED presentations, a responsive and adaptive risk assessment approach is essential. While the current version of IMPACT demonstrates strong retrospective discriminative performance, a prospective real-time deployment would require a revised model restricted to predictors available at the time of triage, notably excluding LoS, which is only known at discharge. Such a triage-time version of IMPACT is identified as a direction for future work. Additionally, clinical utility could be further enhanced by incorporating real-time clinical data streams as they become available during the ED encounter. Integrating continuous monitoring inputs such as vital signs, laboratory results, and ongoing clinical observations would allow the model to dynamically update patient risk profiles as new information becomes available, enabling the early identification of clinical deterioration before critical events occur [43]. Such an enhancement would support a shift from static, one-time risk prediction toward a dynamic, real-time surveillance system within the ED environment. Furthermore, the system’s ability to estimate 10-days and 28-days adverse outcome risks extends its relevance beyond the immediate ED encounter, offering significant value for structuring transitional and post-discharge care planning. Stratifying patients by their risk of short-term adverse outcomes would allow healthcare teams to allocate follow-up resources more effectively, particularly for vulnerable populations such as Māori and Pacific communities, who historically experience higher rates of post-discharge morbidity and mortality.

The methodology demonstrates the potential for broader application across a range of clinical contexts beyond the ED. This approach could be extended into inpatient care pathways, surgical triage systems, and the management of chronic disease populations. This extensibility supports standardised risk assessment throughout the healthcare system, enabling consistent evaluation across departments. Furthermore, the structured nature of IMPACT positions it well for integration with broader healthcare analytics platforms and AI initiatives. Embedding the model within AI-driven ecosystems would allow for continuous refinement of predictive accuracy through the incorporation of real-time data inputs and iterative machine learning algorithms. Maintaining interpretability across these extended applications will remain central to IMPACT’s design, ensuring transparency and accountability as it scales within AI-driven healthcare systems. In addition, prospective testing within live emergency department workflows will be essential to evaluate the model’s real-time performance and clinical impact. These steps will support the full integration of IMPACT into decision-support systems, ensuring its reliability, scalability, and practical value for routine use.

This study has several limitations that should be acknowledged to ensure transparent interpretation of the findings. Although rigorous data cleaning and imputation procedures were applied, data quality issues such as missing values and occasional coding inconsistencies may have influenced the robustness of results. The absence of cause-of-death information limited the ability to attribute mortality events to specific clinical conditions; this will be addressed in future work through the inclusion of Diagnosis-Related Group (DRG) [39] and International Classification Diseases, Tenth Revision (ICD-10) codes [44]. Additionally, certain potentially influential variables, such as socioeconomic status, pre-existing comorbidities, and hospital staffing levels, were not available in the dataset and may have acted as unmeasured confounders. As the analysis was conducted using data from a single tertiary hospital, generalisability to other healthcare settings may be limited. Future studies involving multi-centre validation and more comprehensive variable inclusion will be important to strengthen the model’s applicability and support equitable, system-wide implementation. The imputation of missing WT and LoS values with zero, and missing TS with the lowest urgency category, represents a strong assumption that may introduce systematic bias if missingness is informative (e.g., if patients with missing WT differ systematically from those with recorded values). A formal sensitivity analysis using alternative strategies such as median imputation or multiple imputation by chained equations was not conducted and is identified as a methodological priority for future work. Model calibration has not been formally assessed in this study. Brier scores, calibration curves, and related metrics should be computed and reported in future validation work prior to clinical deployment.

## Conclusion

This study makes several notable contributions to both academic knowledge and healthcare practice. It introduces the IMPACT framework, a risk scoring system that integrates operational, clinical, and demographic factors into a single, interpretable model, advancing beyond traditional single-factor predictors in emergency care.

Beyond confirming known risk factors, IMPACT reframes how emergency department performance and patient outcomes can be evaluated. By translating complex patient and operational data into actionable risk probabilities, IMPACT enables continuous performance assessment, equitable benchmarking, and proactive identification of systemic bottlenecks. The discovery of the inverse relationship between waiting times and adverse outcomes challenges conventional interpretations of ED efficiency metrics and highlights the need for context-sensitive evaluation. The integration of ethnicity within the model allows for systematic equity monitoring, offering a data-driven mechanism to identify and address disparities affecting Māori and Pacifica populations. This capability extends beyond analytics to support policy reform, targeted resourcing, and the development of culturally responsive care strategies.

In its current form, IMPACT is a retrospective risk-stratification and service-evaluation framework rather than a deployed point-of-care tool. With further development, specifically, the framework could form the analytical basis for a future clinical decision-support system embedded within electronic health record workflows. Looking ahead, developing an automated, dynamic system capable of continuously updating individual risk scores will further enhance its predictive utility and responsiveness. Finally, multi-centre validation and prospective testing will ensure the model’s scalability and generalisability, supporting its adoption as a national framework for equitable, data-informed emergency care delivery.

## Data Availability

In accordance with New Zealand’s data sovereignty regulations, including the Privacy Act 2020, the dataset contains sensitive personal information and cannot be publicly shared to safeguard patient privacy. Access is restricted and requires ethical approval and compliance with national data protection standards. Researchers interested in accessing similar data should contact Health New Zealand and follow the necessary ethical and legal procedures.

## Declarations

### Clinical Trial

Clinical trial number: not applicable.
